# Regression of Anabolic-Androgenic Steroid-Associated Cardiomyopathy Eight Years After Cessation of Anabolic-Androgenic Steroid Abuse: A Case Report and Literature Review

**DOI:** 10.3390/jcm15197521

**Published:** 2026-09-27

**Authors:** Pavel S. Baranov, Maria V. Amosova, Marina N. Vukolova, Sergey V. Pirozhkov, Yulia A. Lutokhina

**Affiliations:** 1 Department of Endocrinology, I.M. Sechenov First Moscow State Medical University (Sechenov University), 119991 Moscow, Russia; 2 Professor’s Clinic of Endocrinology and Diabetes LLC, Andreevskaya Naberezhnaya 1, Bldg. 1, 119334 Moscow, Russia; 3 Department of Pathological Physiology, I.M. Sechenov First Moscow State Medical University (Sechenov University), 119991 Moscow, Russia; 4 Department of Internal Medicine No. 1, I.M. Sechenov First Moscow State Medical University (Sechenov University), 119991 Moscow, Russia

**Keywords:** anabolic-androgenic steroids, AAS-associated cardiomyopathy, left ventricular hypertrophy, reverse remodeling, echocardiography, bodybuilding, case report

## Abstract

The non-medical use of anabolic-androgenic steroids (AAS) is widespread among strength-training athletes and, increasingly, among men who have no medical indication for testosterone therapy. The cardiotoxic profile of these drugs includes concentric myocardial hypertrophy, systolic and diastolic dysfunction, myocardial fibrosis and an elevated risk of sudden cardiac death. Whether AAS-associated cardiomyopathy is reversible once AAS are withdrawn remains unsettled: published outcomes range from near-complete recovery to irreversible severe biventricular dysfunction. Here we report the case of a man first assessed at the age of 26, after 33 cumulative months of supraphysiological AAS abuse, and reassessed eight years later. Marked regression of concentric left ventricular hypertrophy, with an accompanying reduction in right ventricular free wall thickness, was documented: left ventricular (LV) mass index fell from 261 to 111 g/m^2^ (57%), interventricular septal and posterior wall thickness decreased by 47% and 49%, and relative wall thickness fell from 0.86 to 0.43. The transmitral E/A ratio rose from 1.18 to 1.45. No cardiovascular pharmacotherapy was given at any stage of follow-up, which distinguishes this observation from previously published cases in which heart-failure-directed treatment was given concurrently.

## 1. Introduction

Anabolic-androgenic steroids (AAS) comprise testosterone and its synthetic derivatives, all of which combine anabolic with androgenic activity. In the meta-analysis by Sagoe et al., the pooled global lifetime prevalence of non-medical AAS use across the included samples was 3.3%, with a corresponding estimate of 6.4% among males [1]. Use is no longer confined to strength athletes. Men who do not compete in any sport increasingly take these drugs to improve their physique, libido, or recovery from physical and mental exertion [2].

The cardiotoxic effects of AAS are well characterized and include concentric left ventricular (LV) hypertrophy, systolic and diastolic dysfunction and myocardial fibrosis, together with an increased risk of coronary atherosclerosis and life-threatening ventricular arrhythmia, including sudden cardiac death [3,4,5]. Several recent studies have sharpened the clinical picture. Among 20,286 competitive bodybuilders followed by Vecchiato et al., all-cause mortality was 81 per 100,000 person-years, and sudden cardiac death accounted for 38% of all deaths; its incidence among professionals exceeded that among amateurs more than fourteen-fold, although the authors caution that the available data do not allow this excess to be attributed to AAS alone, given the contribution of extreme contest preparation, electrolyte disturbance and psychiatric illness [6]. In a Danish nationwide registry study of 1189 sanctioned AAS users and 59,450 age- and sex-matched controls followed for about 11 years, AAS users were close to nine times more likely to develop cardiomyopathy (adjusted hazard ratio 8.90; 95% CI 4.99–15.88) [7]. A meta-analysis of 35 studies (around 2000 men) pooled differences between AAS users and non-users and reported reductions in left ventricular ejection fraction (LVEF) and global longitudinal strain (GLS) alongside increases in septal and posterior wall thickness and in LV mass; that analysis compared users with non-users but did not itself examine a dose-response relationship [8].

Whether these structural and functional changes reverse once AAS are withdrawn remains contested, and the published evidence points in both directions. Several studies argue for recovery. The prospective HAARLEM cohort showed complete normalization of echocardiographic parameters at a median of eight months after a 16-week cycle [3]. The only long-term prospective cohort found that LV mass decreased and LV systolic function appeared to improve in men who discontinued AAS [9]. Myocardial dysfunction was likewise less pronounced in former than in current users in the cross-sectional FIDO-DK study [10]. Other findings point the other way. Systolic and coronary microvascular dysfunction persist for years after cessation in a proportion of former users [11,12], and individual reports range from substantial functional recovery [13,14,15] to irreversible dilated cardiomyopathy [16]. No agreed criteria for predicting reversibility in an individual patient have yet been established [5,17].

Most of what is known about the reversibility of cardiac remodeling after AAS use comes from group-level data. Cohort and cross-sectional studies work with mean values and cannot trace the trajectory of a single patient, while published case reports rarely extend beyond one or two years of follow-up and almost invariably involve concurrent heart failure therapy, which makes it impossible to separate the effect of AAS withdrawal from the effect of treatment [13,14,15,18]. We report here the case of a young man with severe AAS-associated cardiomyopathy following 33 cumulative months of supraphysiological AAS combinations, in whom cardiac structure and function were followed for eight years after withdrawal of all agents, without any cardiovascular pharmacotherapy at any point during follow-up. This setting allows the observed changes to be examined in the absence of heart-failure-directed treatment, and adds an individual, quantitatively documented trajectory to the existing group-level evidence. The case is accompanied by a review of current data on the pathogenesis and reversibility of AAS-associated cardiomyopathy.

## 2. Materials and Methods

We retrospectively reviewed the outpatient records and imaging reports of a single patient examined twice, eight years apart. Data were drawn from the clinical history, physical examination, resting 12-lead electrocardiography and transthoracic echocardiography.

Both echocardiographic studies, the baseline study in 2017 and the follow-up study in 2025, were performed by the same sonographer. The baseline study was performed on a GE Voluson S8 system (GE Healthcare, Chicago, IL, USA) at a private multidisciplinary clinic, using standard cardiac views and the manufacturer’s cardiac measurement package; the transducer type was not documented in the original report. The follow-up study was performed on a Mindray DC-60 Pro system (Mindray Bio-Medical Electronics Co., Shenzhen, China), also by the same sonographer. The patient specifically sought her out to eliminate inter-observer variability, and the change of platform resulted from her having moved to a different institution. Measurements followed the chamber quantification recommendations of the American Society of Echocardiography and the European Association of Cardiovascular Imaging and covered linear and volumetric dimensions of all cardiac chambers, LV mass, systolic and diastolic function, and valve morphology and function. LV mass was recalculated by the authors from the linear dimensions recorded in the original reports of both examinations, using the Devereux formula [LV mass (g) = 0.8 × 1.04 × ((IVS + LVEDD + PW)^3^ − LVEDD^3^) + 0.6, where IVS is interventricular septal thickness, LVEDD is LV end-diastolic diameter and PW is posterior wall thickness, all in cm]; these uniformly derived values are used as the primary longitudinal measure, and the values generated by each system are reported in Section 4.3. Relative wall thickness was calculated as (2 × PW)/LVEDD. LV mass was indexed to body surface area calculated by the DuBois formula.

AAS use, and its subsequent cessation, along with the use of recombinant growth hormone and other pharmacologically active agents, was established from the clinical history. Compounds, doses and durations for all four cycles were reconstructed from contemporaneous personal records retained by the patient rather than from medical documentation and are given in Table 1. The precise sequence in which agents were combined within a cycle could not be reconstructed. Exposure was additionally documented by laboratory testing at five time points between December 2015 and October 2017 and after cessation, covering the gonadal axis, sex hormone-binding globulin, dihydrotestosterone, insulin-like growth factor 1, aminotransferases, the lipid profile, erythrocyte indices and insulin sensitivity (Table 2).

The report was prepared in accordance with the CARE (CAse REport) guidelines. Written informed consent for the analysis and publication of the medical data, including the results of diagnostic investigations, was obtained from the patient. The study was conducted in accordance with the principles of the Declaration of Helsinki.

For the review component, we searched PubMed, Embase, Scopus, and Google Scholar using combinations of the terms “anabolic androgenic steroids”, “steroid cardiomyopathy”, “left ventricular hypertrophy”, “reversibility”, and “echocardiography”. Prospective and cross-sectional clinical studies, systematic reviews, meta-analyses and case reports published in peer-reviewed journals between 2009 and 2026 were considered, with preference given to recent works reflecting the current state of the evidence. The final search was run in June 2026. No formal screening log was maintained, and statements about the novelty of the present case therefore refer to the cases identified in this narrative search rather than to an exhaustive survey. No date restriction was applied to experimental and mechanistic sources, several of which predate this period. The review is narrative rather than systematic.

The manuscript was originally written in Russian. DeepL Write (https://www.deepl.com/write) (DeepL SE, Cologne, Germany, accessed on 28 July 2026) and Claude Opus 4.5 (Anthropic, San Francisco, CA, USA) were used to translate it into English and to adapt the wording to the conventions of English-language academic writing rather than render-ing the Russian text literally. No AI tool was used to collect, analyze or interpret the clinical data. All clinical data, measurement results, references and bibliographical de-tails were identified and analyzed by the authors, who accept full responsibility for the content of this publication.

## 3. Case Presentation

### 3.1. History

A 26-year-old man presented to a cardiologist in December 2017 with palpitations that he described as an irregular heartbeat, occurring mainly as he was falling asleep. He had trained in bodybuilding for the preceding 11 years, with three to six resistance sessions per week. A three-generation family history was obtained and was negative for hypertrophic cardiomyopathy, for sudden cardiac death and for cardiovascular death before the age of 70. He had never previously sought medical attention, did not smoke and did not drink alcohol to excess.

From 2013 onwards, he had used AAS in cycles while preparing for bodybuilding competitions: five months in 2013, seven months in 2014, seven months in 2015, and a continuous 14-month period across 2016 and 2017. Cumulative exposure amounted to 33 months. Drug combinations and doses varied every two to three months; the regimens of all four cycles, with cumulative doses, are listed in Table 1.

Alongside the AAS, the patient used the additional agents listed in Table 3, as well as arginine, citrulline, a multivitamin preparation, fish oil and caffeine.

He sought medical advice both because of the palpitations and because he had come to recognize the potential harm of prolonged AAS use.

### 3.2. Baseline Assessment (December 2017)

On examination, the patient’s height was 170 cm and his weight 100 kg (body mass index 34.6 kg/m^2^), with body fat of 11–12% on bioimpedance analysis. He appeared well. The skin was unremarkable, with no cyanosis or peripheral edema. Breath sounds were vesicular at a respiratory rate of 15/min, with no crackles. Heart sounds were normal but irregular because of ectopic beats, at a rate of 64/min, with no murmurs. Blood pressure was 140/85 mm Hg. The liver was not enlarged, the spleen was not palpable, and the abdomen was soft and non-tender.

A resting 12-lead electrocardiogram showed sinus rhythm at 62/min with occasional ventricular ectopic beats and voltage criteria for LV hypertrophy.

Transthoracic echocardiography demonstrated severe concentric LV hypertrophy, with an interventricular septum of 22 mm and an LV posterior wall of 21 mm, and an LV mass of 551 g calculated by the Devereux formula, more than 2.4 times the upper reference limit for men. The right ventricular free wall measured 8.2 mm in the parasternal long-axis view; the cardiac phase was not specified in the original report, and no right ventricular functional parameters (TAPSE, S′, fractional area change or strain) were recorded. Right atrial enlargement was based on two linear dimensions in the apical four-chamber view rather than on indexed area or volume. LV end-systolic diameter was 16 mm. The inferior vena cava measured 20 mm with more than 50% inspiratory collapse, indicating normal right atrial pressure. Regional LV wall motion was normal. The transmitral E/A ratio was 1.18, which is below the range expected in a man of this age. Septal and lateral e′, E/e′, left atrial volume index, and tricuspid regurgitation velocity were not recorded, so diastolic function could not be graded by current criteria [19], and only the transmitral filling pattern is reported here. Moderate pulmonary regurgitation was present, corresponding to the historical grading used in the original report; the method by which severity was assessed is not documented. There was no evidence of pulmonary hypertension. Full baseline data alongside the follow-up study are given in Table 4.

### 3.3. Management and Follow-Up

Given the absence of heart failure symptoms, preserved LV systolic function, normal regional wall motion and only isolated ventricular ectopic beats on the resting electrocardiogram, we elected to observe rather than to start cardiovascular pharmacotherapy. The patient was strongly advised to stop all AAS, recombinant growth hormone and the accompanying pharmacologically active agents.

He did so in December 2017, discontinuing AAS, recombinant growth hormone, clenbuterol and yohimbine at the same time. The palpitations resolved spontaneously over the following two to three months, between December 2017 and March 2018, without antiarrhythmic treatment. He subsequently modified his lifestyle: resistance training continued but at reduced volume and intensity, moderate aerobic exercise was added, and he maintained a balanced diet. Lean body mass declined gradually over the following years.

No repeat echocardiography was performed between 2017 and 2025. The patient reported that AAS, growth hormone and the other agents were not resumed. Annual electrocardiography showed voltage criteria for hypertrophy until 2020, after which they were no longer present. Beyond the isolated ventricular ectopic beats present at baseline, no sustained ventricular arrhythmia was documented on any of the available recordings. At review in October 2025, he described a considerable improvement in general wellbeing over the intervening years, with better work capacity, greater endurance and improved tolerance of exercise of varying types and intensity as lean mass fell and his lifestyle changed. He reported no cardiac symptoms.

### 3.4. Findings at Eight Years (October 2025)

By this time, the patient was 34 years old. His weight had fallen to 80 kg at the same height of 170 cm (body mass index 27.7 kg/m^2^), while body fat remained 11–12% on bioimpedance analysis. He appeared well, with no cyanosis or peripheral edema. Breath sounds were vesicular at a respiratory rate of 16/min. Heart sounds were normal and regular at 70/min, with no murmurs, and blood pressure was 120/75 mm Hg. Abdominal examination was unremarkable.

Laboratory testing in July 2026 showed recovery of the hypothalamic-pituitary-gonadal axis, normalization of the lipid profile and of aminotransferase activity, and resolution of the erythrocytosis and insulin resistance documented during exposure (Table 2). The full clinical timeline of the case, including exposure, investigations, and clinical course, is summarized in Table 5.

The electrocardiogram showed sinus rhythm at 56/min and was otherwise normal, with no voltage criteria for hypertrophy, arrhythmia, conduction abnormality or ischemic change (Figure 1).

Repeat transthoracic echocardiography, performed eight years after the baseline study, showed a convincing improvement in cardiac structure and function following cessation of AAS and in the absence of any cardiovascular pharmacotherapy. The results of the two studies are compared in Table 4.

The most striking finding was regression of LV hypertrophy. Septal thickness fell from 22 to 11.7 mm (47%) and posterior wall thickness from 21 to 10.8 mm (49%). Calculated by the Devereux formula for both examinations, LV mass fell from 551 to 214 g (61%); with the accompanying reduction in body weight from 100 to 80 kg, LV mass index decreased from 261 to 111 g/m^2^ (57%), that is, from more than twice the upper reference limit for men to within normal limits. Relative wall thickness decreased from 0.86 to 0.43, indicating marked regression of concentric remodeling, with a residual value that remains marginally above the threshold of 0.42. The reduction in body weight was predominantly attributable to loss of fat-free mass. The right side of the heart changed in parallel: right ventricular free wall thickness fell from 8.2 to 3.9 mm (52%), and right atrial dimensions in the apical four-chamber view from 50 × 57 to 44 × 47 mm.

LVEF at follow-up, measured by the biplane Simpson method, was 75%. The value of 90% recorded in 2017 was obtained by the Teichholz method, which performs poorly when LV geometry is distorted and which, in the presence of concentric hypertrophy and a very small systolic cavity dimension (end-systolic diameter 16 mm), overestimates ejection fraction. Because the two values were obtained by different methods on different platforms, the change from 90% to 75% cannot be interpreted as a transition from hyperkinetic to normal hemodynamics, and no conclusion about longitudinal change in systolic function can be drawn.

The transmitral E/A ratio rose from 1.18 to 1.45, a value consistent with the age-specific reference range. Because comprehensive assessment of diastolic function was unavailable at either time point, this is reported as a change in the transmitral filling pattern rather than as normalization of diastolic function.

Among the valves, the pulmonary regurgitation seen in 2017 was no longer detected; pulmonary artery systolic pressure was 20 mm Hg, with no evidence of pulmonary hypertension. Since the two examinations were performed on different systems, whose color Doppler sensitivity and settings are not comparable, this change is reported as an observation rather than as evidence of reverse remodeling. Several thickened false tendons were visualized within the LV cavity; as these are congenital structures, they were in all likelihood already present at the baseline examination.

Taken together, these findings document marked regression of the structural changes of AAS-associated cardiomyopathy over eight years of reported abstinence from AAS and the other agents used, in the absence of any cardiovascular pharmacotherapy.

## 4. Discussion

In this young man, who had abused supraphysiological doses of AAS over a prolonged period, concentric left ventricular hypertrophy regressed markedly within eight years of cessation, with an accompanying reduction in right ventricular free wall thickness, and the transmitral filling pattern changed in parallel. This occurred without any cardiovascular pharmacotherapy at any stage, which distinguishes the observation from previously published cases in which heart-failure-directed treatment was given concurrently. That fact is worth setting against current understanding of how AAS-associated cardiomyopathy develops and whether it resolves.

The attribution of the baseline phenotype and of its regression to AAS alone requires caution. When AAS were stopped, the patient simultaneously discontinued recombinant growth hormone, clenbuterol and yohimbine, reduced the volume and intensity of resistance training, introduced moderate aerobic exercise and lost 20 kg of body weight, while arterial pressure fell from 140/85 to 120/75 mm Hg [20]. Growth hormone deserves particular attention, and its exposure is documented here: insulin-like growth factor 1 was elevated at 569.4 and 376.2 ng/mL (reference 121–336) in August and October 2017, with concurrent insulin resistance (HOMA-IR 7.3), and supraphysiological growth hormone is recognized to promote concentric remodeling. Three observations nevertheless suggest that its contribution was not the dominant one. Cumulative AAS exposure of 33 months was 2.75 times longer than the 12 months of growth hormone use, and no growth hormone was used during the 2013 or 2016 cycles. The published cases of severe or irreversible AAS-associated cardiomyopathy discussed below involved no concomitant growth hormone use, indicating that AAS alone can produce a more severe phenotype than the one described here; we are not aware of a comparable phenotype attributed to isolated growth hormone use in an athlete, and the EAPC position statement notes that little is known about the direct cardiovascular effects of excessive growth hormone administration in this population [20]. Clenbuterol and yohimbine, whose elimination half-lives are approximately 35 h and one hour, do not accumulate, and the cardiovascular effects attributed to them are acute rather than hypertrophic [20]. The effects of AAS, growth hormone, the reduction in training load and the change in body composition cannot be separated on the available data.

Four alternative explanations for the baseline phenotype deserve comment, and none can be definitively excluded on the available data.

Sarcomeric hypertrophic cardiomyopathy can present with a septal thickness of 22 mm in a man of this age. Against it are a three-generation family history negative for hypertrophic cardiomyopathy, the complete resolution of electrocardiographic abnormalities, which characteristically persist in sarcomeric disease; the absence of sustained ventricular arrhythmia over eight years; and, most importantly, the regression itself, since sarcomeric hypertrophy does not resolve spontaneously. The assessment followed the multiparametric approach to etiology and phenocopies set out in the 2023 ESC Guidelines, in which pedigree analysis, the 12-lead electrocardiogram, laboratory markers and serial imaging are core diagnostic instruments [21].

Physiological remodeling of the athlete’s heart is the second alternative, and the patient had trained in bodybuilding for 11 years. Wall thickness in strength-trained athletes rarely exceeds 15 mm, and biventricular values of 22 and 21 mm lie far outside that range, so physiological remodeling does not account for the degree of hypertrophy observed; a training-related component superimposed on the pharmacological one cannot be excluded. Resistance training continued throughout follow-up, at reduced volume and intensity, while the hypertrophy regressed.

Growth-hormone-related hypertrophy is the third, and its exposure is documented by the elevated insulin-like growth factor 1 concentrations reported above. For the reasons set out earlier, we consider its contribution possible but unlikely to have been dominant; it cannot, however, be separated from the androgenic component on the available data.

Hypertension is the fourth. Arterial pressure of 140/85 mm Hg was recorded at a single examination during active AAS use, when sodium and water retention would be expected; sustained hypertension was not documented either before or after, and pressure was 120/75 mm Hg at follow-up without antihypertensive treatment. A single measurement is nevertheless insufficient either to establish or to exclude a hypertensive contribution. Cardiac magnetic resonance imaging would have resolved much of this uncertainty: it quantifies ventricular mass without the geometric assumptions on which linear measurements depend, and late gadolinium enhancement with T1 and extracellular volume mapping would have identified residual myocardial fibrosis and assisted in distinguishing toxic remodeling from a sarcomeric phenotype. It was not available in this case and should be considered a standard component of assessment in future reports of this kind.

### 4.1. Pathophysiology of AAS-Associated Cardiomyopathy

AAS injure the heart through a combination of direct, receptor-mediated actions on cardiomyocytes and vascular cells and indirect effects mediated by neurohormonal activation, hemodynamic load and systemic inflammation [5,22,23]. These mechanisms are worth reading against the findings in this patient, since several of them account not only for how the cardiomyopathy developed but also for why so much of it proved reversible.

*Direct receptor-mediated effects on the cardiomyocyte.* The sequence begins with androgen receptors, which are expressed in cardiomyocytes of both sexes and in human as well as animal myocardium. Marsh et al. demonstrated androgen receptor transcripts in human and rodent heart and showed that testosterone and dihydrotestosterone both produce a receptor-specific hypertrophic response in cultured myocytes, measured as [3H]phenylalanine incorporation into myocyte protein and as atrial natriuretic peptide secretion [24]. Androgen receptors are also present in vascular endothelium, where testosterone and dihydrotestosterone regulate endothelial nitric oxide synthase, plasminogen activator inhibitor 1 and tissue plasminogen activator [25].

Two signaling routes operate in parallel. The genomic route acts through androgen response elements to upregulate transcription of contractile protein genes. The non-genomic route is faster and calcium-dependent. Testosterone raises intracellular Ca^2+^ in cultured rat cardiac myocytes within minutes by a mechanism that does not require gene transcription [26]. Downstream of this, Altamirano et al. showed that testosterone increases phosphorylation of mTOR and of its targets S6K1 and 4E-BP1 through the IP3/Ca2+ and MEK/ERK1/2 pathways and that inhibiting mTOR abolishes the increases in β-myosin heavy chain and α-skeletal actin gene expression, in cell size and in amino acid incorporation that testosterone otherwise produces [27]. Sustained supraphysiological exposure therefore drives a coordinated transcriptional and translational program of concentric hypertrophy. The baseline study in this patient showed that program at an extreme, with septal and posterior wall thickness of 22 and 21 mm and an LV mass index of 261 g/m^2^.

*Neurohormonal activation and hemodynamic load.* Androgen receptors are not the only driver of pathological myocardial hypertrophy. AAS raise sympathetic tone, stimulate renin release and increase aldosterone secretion, producing sodium and water retention and a rise in arterial pressure [22,28,29]. Aldosterone acting on the mineralocorticoid receptor promotes myocardial reactive oxygen species generation, inflammation and fibrosis independently of its effect on pressure, and AAS additionally upregulate angiotensin II type 1 receptors in cardiac tissue and the vessel wall [22,23]. The pressure-dependent and pressure-independent routes to hypertrophy therefore operate together rather than as alternatives. Both routes are potentially relevant here. Arterial pressure at presentation was 140/85 mm Hg and was 120/75 mm Hg at follow-up without antihypertensive treatment. Sodium and water retention, circulating blood volume, preload and afterload were not measured, so the proposition that part of the hypertrophic stimulus was load-related, and diminished once that load was removed, is offered as a hypothesis consistent with the observed change in pressure rather than as a patient-level finding. The concurrent reduction in body weight, which was predominantly attributable to loss of fat-free mass, is an equally plausible contributor.

*Endothelial dysfunction and vascular inflammation.* At physiological concentrations, androgen receptor activation in endothelium increases nitric oxide synthesis through the MAPK and PI3K/Akt pathways and enhances prostacyclin release, both of which favor vasodilatation [23,25]. Supraphysiological exposure reverses this. Nitric oxide bioavailability and prostacyclin release fall and endothelium-dependent vasodilatation is impaired [23,30]; in young AAS users, carotid reactivity and flow-mediated dilatation are both significantly reduced compared with weightlifting controls [31]. High androgen concentrations also shift the endothelium toward a proinflammatory phenotype, with increased expression of VCAM-1, ICAM-1 and E-selectin and greater monocyte adhesion [32,33]. The clinical reach of this extends beyond blood pressure, since it links AAS use both to accelerated coronary atherosclerosis and to the coronary microvascular dysfunction that Bulut et al. found to persist for years after cessation [12].

*Oxidative stress, apoptosis and myocyte loss.* Supraphysiological AAS disrupt redox balance in the myocardium. In mice, AAS impair both mitochondrial function and redox status in cardiac tissue [34]. In rats, nandrolone decanoate increases NADPH oxidase activity in the heart, lowers antioxidant enzyme activity and raises protein carbonyl content [35]. Amini et al. found that nandrolone raised cardiac 8-hydroxy-2′-deoxyguanosine, cytochrome c, p53 and the ratio of cleaved to pro-caspase-3, increased the number of TUNEL-positive cardiomyocytes and activated the TLR4/NF-κB/NLRP3 axis, all of which N-acetylcysteine partly reversed [36]. Cardiomyocyte apoptosis under AAS exposure has been reproduced across agents and models, from adult rat ventricular myocytes treated with commonly abused steroids [37] to testosterone- and stanozolol-treated hearts, in which hypertrophy, fibrosis and apoptosis appear simultaneously [38,39,40]. Myocyte loss on this scale is what converts reversible hypertrophy into fibrous replacement, and it is the most plausible substrate for the irreversible component of the disease.

*Autophagy: an inference rather than a finding.* One element of this sequence is often reported with more confidence than the evidence supports, and we state it here with the qualification it requires. The anabolic signaling that AAS activate, in particular the Akt/mTOR axis, is known to suppress autophagy in models of cardiac hypertrophy unrelated to AAS, and testosterone has been shown to activate mTOR in cardiomyocytes [27]. Whether AAS impair autophagy in the heart directly has not been demonstrated, and conclusive experimental data are lacking [23]. The proposed downstream consequence, defective clearance of damaged mitochondria and of protein aggregates, is therefore biologically plausible but remains unproven.

*Experimental confirmation at the level of the whole organ.* These cellular mechanisms converge on a phenotype that has been reproduced in vivo. Moraes et al. treated rats with nandrolone decanoate in combination with resistance training (RT) and compared them with animals given nandrolone alone. The combined treatment (N-T) increased LV diameter by 25%, right ventricular diameter by 33% and septal thickness by 41.7%, and reduced LVEF by 12.3% compared with nandrolone alone. Relative to trained controls, the combination also reduced expression of the calcium-handling proteins ryanodine receptor type 2 and total phospholamban, and increased myocardial malondialdehyde [41]. Tellingly, the remodeling remained maladaptive even though the contractility of isolated cardiomyocytes in this group was enhanced, which illustrates how far cellular contractile performance can diverge from integrated pump function under AAS exposure.

*From remodeling to the arrhythmogenic substrate.* AAS-associated hypertrophy frequently outpaces angiogenesis, producing relative capillary insufficiency and chronic subendocardial ischemia even when the epicardial coronary arteries are intact. The interstitial and perivascular fibrosis that follows, together with the myocyte loss described above, slows conduction and makes refractoriness spatially heterogeneous, establishing the anatomical conditions for re-entry arrhythmia [5,23]. Autonomic changes compound this, since AAS users show reduced heart rate variability and a shift toward sympathetic predominance [42]. AAS act on cardiomyocyte electrophysiology directly as well, modulating voltage-gated ion channels, principally IKr and IKs, which produces heterogeneous QT prolongation and raises the risk of ventricular tachyarrhythmia and sudden cardiac death [5,23]. Right-sided involvement belongs to the same process rather than being incidental to it: Kasikcioglu et al. showed that AAS impair right ventricular as well as left ventricular function [43], and in the present patient, right ventricular free wall thickness was 8.2 mm at baseline and 3.9 mm at follow-up.

### 4.2. Clinical and Imaging Manifestations

Drawing on current prospective and cross-sectional studies and meta-analyses, the myocardial changes associated with prolonged supraphysiological AAS use include the following:

- *Concentric LV hypertrophy*, with increases in LV mass and in septal and posterior wall thickness. The degree of hypertrophy relates to cumulative exposure in studies that analyzed weekly dose or duration of use, including the prospective HAARLEM cohort [3] and the cross-sectional study by Baggish et al. [4]. In the pooled between-group meta-analysis by Cavalcante et al., septal thickness increased by a mean of 1.24 mm (95% CI 0.79–1.69; *p* < 0.01) and posterior wall thickness by 1.09 mm [8].
- *LV diastolic dysfunction*, with impaired relaxation and a fall in the E/A ratio. In HAARLEM, the E/A ratio declined by 0.45 (95% CI −0.69 to −0.21; *p* < 0.001) between the start and the end of the cycle and returned to baseline after the recovery period [3]; diastolic dysfunction has also been documented in former users of AAS [4,11].
- *Reduced LV systolic function* manifests first as a fall in global longitudinal strain (GLS) and, in more severe cases, as a fall in LVEF. Abdullah et al. studied 101 AAS users with a mean duration of use of 11 ± 7 years and found a mean LVEF of 49 ± 7% against 59 ± 5% in controls (*p* < 0.001) [11]. In the meta-analysis by Cavalcante et al., AAS use was associated with a mean reduction in LVEF of 2.25% (95% CI −3.41 to −1.09; *p* < 0.01) and a worsening of GLS of 3.34% (95% CI 2.93–3.76; *p* < 0.01) [8].
- Biventricular involvement, with right ventricular wall thickening and functional impairment on speckle-tracking echocardiography and cardiac magnetic resonance imaging [11,43,44]. In FIDO-DK, cumulative AAS exposure was independently associated with worsening of GLS in both the left (regression coefficient 0.08; 95% CI 0.03–0.12; *p* = 0.002) and the right ventricle (0.08; 95% CI 0.03–0.13; *p* = 0.001) [10].
- Myocardial fibrosis, seen as late gadolinium enhancement on cardiac magnetic resonance imaging, predominantly in the interventricular septum in an intramyocardial pattern. Fibrosis has also been confirmed at autopsy in cases of sudden cardiac death among AAS users, where severe LV hypertrophy and cardiomegaly were accompanied by myocardial fibrosis and necrosis [6,45].
- *Accelerated coronary atherosclerosis*, associated with endothelial dysfunction, dyslipidemia (reduced HDL and raised LDL cholesterol) and a higher coronary artery calcium score. Baggish et al. found coronary plaque volume in AAS users to be significantly greater than in controls and to correlate with duration of use [4]. In FIDO-DK, each additional year of cumulative use raised the odds of a positive coronary calcium score (OR 1.23; 95% CI 1.09–1.39; *p* = 0.001), and exposure beyond five years was associated with more severe calcification [10].
- *Coronary microvascular dysfunction* persists for several years after AAS are stopped. Using ^82^Rb positron emission tomography, Bulut et al. found myocardial flow reserve in former users to be significantly lower than in controls [12].
- Arrhythmia and an increased risk of sudden cardiac death arise from myocardial fibrosis, QT prolongation, autonomic imbalance and the direct proarrhythmic activity of AAS [5,6,23,42].

### 4.3. Reversibility of AAS-Associated Cardiomyopathy: Comparison with Published Data

Evidence on reversibility comes from two kinds of sources: cohort and cross-sectional studies working with group-level measures, and individual case reports. The key studies are summarized in Table 6 and the published cases in Table 7.

The strongest evidence that reverse remodeling can be durable comes from the prospective cohort of Fyksen et al., in which 32 men using AAS and 13 strength-trained non-users, with use and non-use verified by blood and urine analyses, were examined at baseline and again after a median of 16 years [9]. At baseline, the users had taken AAS for a median of 5–10 years and had greater LV mass and lower LVEF than the non-users. By the second examination, 15 had stopped using AAS, a median of 12 years earlier [46], and 17 had continued. In those who had stopped, LV mass had decreased to values similar to those of the non-users, and LVEF had changed by a median of +3%, against −2% in those who continued; the authors describe systolic function as having seemed to improve, including after more than a decade of use [9]. These group-level observations are consistent with the trajectory traced here in an individual and make it less likely that it represents an isolated anomaly or a measurement artifact, although the cohort was small and observational.

The present case makes no claim to the longest follow-up; that of Fyksen et al. is twice as long [9]. Its value lies elsewhere, and rests on three points that Table 7 makes plain. First, it provides an individual, quantitatively documented trajectory of specific echocardiographic measurements, whereas cohort studies work with group means and cannot show the magnitude of regression in a single patient. Second, among the cases identified in our search, this is the only one in which the patient received no cardiovascular pharmacotherapy at any point during follow-up. Patients described by Milevski, Charaya, Angelini and others were all treated with optimal medical therapy for heart failure, which makes it impossible to separate the contribution of AAS withdrawal from that of treatment [13,14,15,16,18]. The present case therefore offers an uncommon opportunity to observe the course of the condition after exposure is removed and in the absence of heart-failure-directed treatment. Third, the baseline burden was substantial, with an LV mass index of 261 g/m^2^, more than twice the upper reference limit, which makes the extent of regression all the more instructive.

One methodological point deserves comment. The two ultrasound systems reported LV mass using their respective built-in algorithms, giving 552 g in 2017 and 240 g in 2025. Because these values are not derived by a common method, we have used the Devereux formula applied to the linear dimensions of both examinations as the primary longitudinal measure, giving the values reported above. On the uniformly calculated values the mass index returned to within the normal range for men, whereas on the system-reported values it remained marginally above it; the difference lies within the intersystem variability described for echocardiographic mass estimation.

The case reported by Charaya et al. is of particular interest [18]. A 49-year-old bodybuilder with more than four years of AAS use initially presented with severe heart failure, an LVEF of 20% and marked LV hypertrophy. After AAS were stopped and optimal medical therapy was started, LVEF recovered to 62% and remained stable for several years. When the patient discontinued all medication on his own initiative and resumed AAS, LVEF fell again to 31%; cardiac magnetic resonance imaging then showed intramyocardial fibrosis in the septum and a transmural scar in the anterior wall, and coronary angiography revealed multivessel disease that ultimately required implantation of a cardioverter-defibrillator [18]. The case illustrates two features of the condition at once: the myocardium retains a considerable capacity for reverse remodeling provided AAS are stopped completely, yet AAS can also induce an irreversible component that becomes apparent when use is resumed. Our finding that regression was sustained across eight years of reported abstinence supports the first of these statements and points to the importance of abstinence being maintained.

The published outcomes are nonetheless strikingly heterogeneous. Angelini et al. described a 31-year-old bodybuilder who had used testosterone esters for almost 12 years and developed severe dilated cardiomyopathy with an LVEF of 23% that did not regress despite complete withdrawal and optimal medical therapy [16]. Additional factors may well have determined that outcome, such as mutations in cardiomyopathy-associated genes or intercurrent myocarditis. At group level, persistent biventricular systolic dysfunction in former users at 6 ± 6 years after cessation was shown by Abdullah et al. [11], and persistent coronary microvascular dysfunction years after cessation by Bulut et al. [12]. Together, these findings indicate that AAS-associated cardiomyopathy has a potentially irreversible component, with myocardial fibrosis and microvascular remodeling as its likely substrate, consistent with the apoptotic myocyte loss described in Section 4.1.

Comparing these studies suggests that cumulative AAS exposure may be one of the factors separating favorable from unfavorable outcomes. In FIDO-DK, among men with more than five years of cumulative use, 35 of 36 had LV mass above and LVEF below the median of the reference range [10]; that observation describes a shift within the normal distribution rather than a pathological threshold, and the five-year cut-off was itself derived in exploratory analyses of coronary calcification. Our patient had 33 cumulative months of use, whereas the patients with irreversible disease described by Angelini and by Abdullah had substantially longer exposure [11,16]. This comparison is descriptive and is not offered as a prognostic threshold.

### 4.4. Factors Favoring the Outcome in the Present Case

Against the range of published findings, several factors plausibly account for the favorable outcome in this patient.

*Age.* He was 26 at the baseline assessment and 34 at follow-up. In the series of Abdullah et al., former users with persistent systolic dysfunction were around 40 years old on average, and the patient with irreversible dilated cardiomyopathy described by Angelini et al. was 31, though with 12 years of cumulative use [11,16]. Younger age and shorter exposure may leave a greater residual capacity for reverse remodeling, through preserved cardiomyocyte functional reserve and less extensive irreversible fibrosis.

*Cumulative AAS exposure.* The extent and reversibility of structural changes relate to the duration and intensity of use in HAARLEM, in the work of Baggish et al. and in FIDO-DK [3,4,10]. Cumulative exposure here was 33 months, which is considerably shorter than the mean duration in the study of Abdullah et al. (11 ± 7 years) and in the case of Angelini et al. (12 years) [11,16]. Cumulative doses over this period amounted to approximately 73 g of testosterone esters, 40 g of boldenone undecylenate, 21.7 g of drostanolone propionate, 13.6 g of stanozolol, 8.7 g of oxandrolone, 7.6 g of metenolone enanthate and 0.5 g of fluoxymesterone (Table 1). Although the cumulative dose was substantial, the exposure was concentrated into a shorter period than in those series, which may explain why the extensive apoptotic myocyte loss and fibrosis that underlie irreversible remodeling did not develop.

*Absence of other cardiovascular risk factors.* The patient had no family history of cardiomyopathy, no long-standing hypertension, dyslipidemia or disordered glucose metabolism; he did not smoke, did not drink alcohol to excess and had no known or clinically evident coronary artery disease. No coronary computed tomography angiography, invasive angiography, stress imaging or perfusion study was performed, so subclinical coronary atherosclerosis cannot be excluded; during exposure the lipid profile was markedly atherogenic, with HDL cholesterol between 0.39 and 0.50 mmol/L and an atherogenic index between 6.9 and 11.6 across five time points, before normalizing after cessation (Table 2). No regional wall motion abnormality was found at follow-up, which argues indirectly against post-ischemic change. Concomitant cardiovascular risk factors worsen the prospects for reverse remodeling in cardiomyopathy of any cause. The contrast with the case of Charaya et al., in which a 49-year-old man developed multivessel coronary disease after resuming AAS, points to how much these additional factors matter [18].

*Cessation of all agents. Unlike the patient of Charaya* et al., who resumed AAS after several years of stable recovery [18], this patient reported stopping all AAS, recombinant growth hormone and the accompanying agents (clenbuterol, yohimbine) outright, and reduced the volume and intensity of his resistance training at the same time; he also ceased competitive bodybuilding at the end of 2017 and did not return to it. Abstinence over the subsequent eight years rests on the clinical history. Intermittent hormonal testing between 2018 and 2026 was reported as within reference ranges on each occasion, although the individual reports are no longer available, and the normal gonadotrophin and testosterone concentrations documented after cessation establish recovery of the gonadal axis at that time without verifying uninterrupted non-use throughout the interval. Withdrawal of androgenic stimulation of cardiomyocyte androgen receptors plausibly removed the hypertrophic and anabolic signal described in Section 4.1, and regression of hypertrophy followed; this sequence is a mechanistic hypothesis consistent with the observation rather than a demonstrated causal chain. The cohort of Fyksen et al. described in Section 4.3 points in the same direction [9], but given its observational design and small size, it shows an association between discontinuation and improvement, and a contribution from duration, dose or other confounders cannot be excluded.

*Hemodynamic changes after withdrawal.* Arterial pressure fell from 140/85 to 120/75 mm Hg between the two assessments without antihypertensive treatment. Sodium and water retention, circulating blood volume, preload and afterload were not measured, so the proposal that reduced volume loading lowered ventricular wall stress and favored reverse remodeling is offered as a hypothesis consistent with the observed change in pressure rather than as a patient-level finding. The concurrent 20 kg reduction in body weight was predominantly attributable to loss of fat-free mass, so the mechanisms by which weight loss reduces ventricular mass in obesity do not apply directly; a reduction in lean body mass may nonetheless contribute, although it would not account for reductions of 47 to 49% in wall thickness following a 20% reduction in body weight. A similar pattern is visible in FIDO-DK, where systolic blood pressure in former users of AAS was significantly lower than in current users [10].

*Lifestyle modification.* The patient took regular moderate-intensity aerobic exercise, maintained a balanced diet and reduced his training load to a level his body could accommodate. The cardioprotective effects of regular aerobic exercise, including improved endothelial function, greater nitric oxide bioavailability and reduced sympathetic activity, may have contributed further to the recovery of cardiac structure and function. Conversely, in the experimental work of Moraes et al., combining nandrolone with intensive resistance training aggravated maladaptive remodeling relative to the drug alone [41], which indirectly supports the value of reducing training intensity in this patient.

The present case therefore belongs to the group of observations with a favorable reverse-remodeling phenotype after AAS withdrawal, forming one pole of the clinical spectrum described in the literature (Table 7). At the opposite pole lie cases of irreversible biventricular cardiomyopathy, characterized by longer cumulative androgen exposure, resumption of AAS, older age and concomitant cardiovascular risk factors. The divergence in outcomes argues for a stratified approach to prognosis in former AAS users, and for working criteria that would identify in advance those patients most likely to undergo reverse remodeling.

### 4.5. Implications for Clinical Practice

This observation has practical implications for the management of patients who abuse AAS. Achieving complete cessation is far from straightforward. Kanayama et al. found that around 30% of AAS users meet criteria for dependence, and that the risk of developing it rises with the duration of use and with starting at a young age [47]. Current expert recommendations stress the need for a non-judgmental diagnostic approach to these patients and set out two parallel strategies, supporting complete cessation when achievable, and minimizing harm when patients are not ready to stop [48]. Magnolini et al. describe how such a strategy works in routine clinical practice [49]. Showing a patient objective, documented regression of structural cardiac change can be a powerful motivator for sustained abstinence. Stopping AAS early, while the patient is still young and cumulative exposure remains limited, appears to be a critical intervention. Neither the present case nor the studies summarized in Table 6 establishes an evidence-based interval for serial imaging or demonstrates benefit from universal echocardiographic screening, and we therefore suggest cardiovascular assessment guided by exposure history, symptoms, arterial pressure, electrocardiographic findings and other cardiovascular risk factors, with echocardiography directed by those findings rather than applied routinely to all former users. A history of AAS use should nonetheless be taken into account even in the absence of symptoms, since impaired left ventricular global longitudinal strain has been described in former users years after cessation [20]. The presentation in this case is consistent with that approach: the patient presented with palpitations, and the hypertrophy was identified in the course of an assessment prompted by that symptom.

The baseline phenotype also invites consideration of ventricular-arterial coupling. Elevated arterial pressure, marked concentric hypertrophy and a hyperdynamic small-cavity physiology with an end-systolic diameter of 16 mm together describe a ventricle operating against increased arterial load with limited stroke volume reserve, a pattern associated with maladaptive ventricular-arterial coupling and detectable with indices combining arterial stiffness and longitudinal strain [50,51]. Neither arterial stiffness nor global longitudinal strain was measured here; the supporting evidence derives from hypertensive rather than AAS-exposed populations, and prospective multicenter validation of such indices is still awaited [51]; this therefore remains a direction for future study rather than an interpretation of the present findings.

### 4.6. Limitations

The retrospective collection of data on AAS regimens does not exclude inaccuracy in the estimation of doses and duration of exposure; regimens were reconstructed from contemporaneous personal records rather than medical documentation, and the sequence in which agents were combined within each cycle could not be established. The concurrent withdrawal of recombinant growth hormone, the reduction in training load and the 20 kg reduction in body weight mean that the regression cannot be attributed to AAS cessation in isolation. Abstinence rests on the clinical history and was not verified by serial toxicological testing. Neither cardiac magnetic resonance imaging nor genetic testing was performed; although the regression itself argues strongly against a sarcomeric etiology, the diagnosis was not formally excluded by these methods, and residual myocardial fibrosis could not be assessed. The absence of interim echocardiography between 2017 and 2025 means that neither the point at which reverse remodeling began nor its rate can be established. Different ultrasound systems were used at baseline and follow-up, and LVEF was obtained by different methods at the two time points, so systolic function is not comparable across the examinations; the use of a single sonographer reduces but does not remove this limitation, and the transducer type used in 2017 is not documented. No digital image archive survives from either examination, so the measurements could not be repeated by a single standardized method, and representative paired images cannot be presented. The original 2017 electrocardiogram is not available and the voltage criterion applied was not recorded; Holter monitoring was never performed, so intermittent arrhythmia cannot be excluded. Comprehensive assessment of diastolic function was not possible at either time point, since tissue Doppler indices, left atrial volume index and tricuspid regurgitation velocity were not recorded; in routine practice in 2017 the assessment of diastolic function frequently rested on the transmitral inflow pattern alone, and the retrospective nature of this observation means the gap cannot now be filled [19]. Only the change in the transmitral filling pattern is therefore reported. Finally, without functional or perfusion imaging, we cannot exclude persistent coronary microvascular dysfunction of the kind described by Bulut et al. in former users [12], nor subclinical coronary atherosclerosis, the likelihood of which is raised in anyone with a history of AAS use [7,10].

## 5. Conclusions

We have described the regression of structural cardiac changes that had developed in a young man abusing supraphysiological doses of anabolic-androgenic steroids.

The defining feature of this observation is the complete absence of cardiovascular pharmacotherapy throughout follow-up, which distinguishes it from previously published cases in which heart-failure-directed treatment was given concurrently. The regression followed cessation of AAS together with the concomitant agents and substantial lifestyle change, and the contribution of each component cannot be separated. Taken together, the available evidence suggests that sustained cessation at a young age, where cumulative exposure has been relatively limited, and there is no concomitant cardiovascular disease, is associated with favorable conditions for the recovery of cardiac structure. Further long-term follow-up studies using contemporary imaging will be needed to define the predictors of reversibility in AAS-associated cardiomyopathy and to inform clinical recommendations.

## Figures and Tables

**Figure 1:**
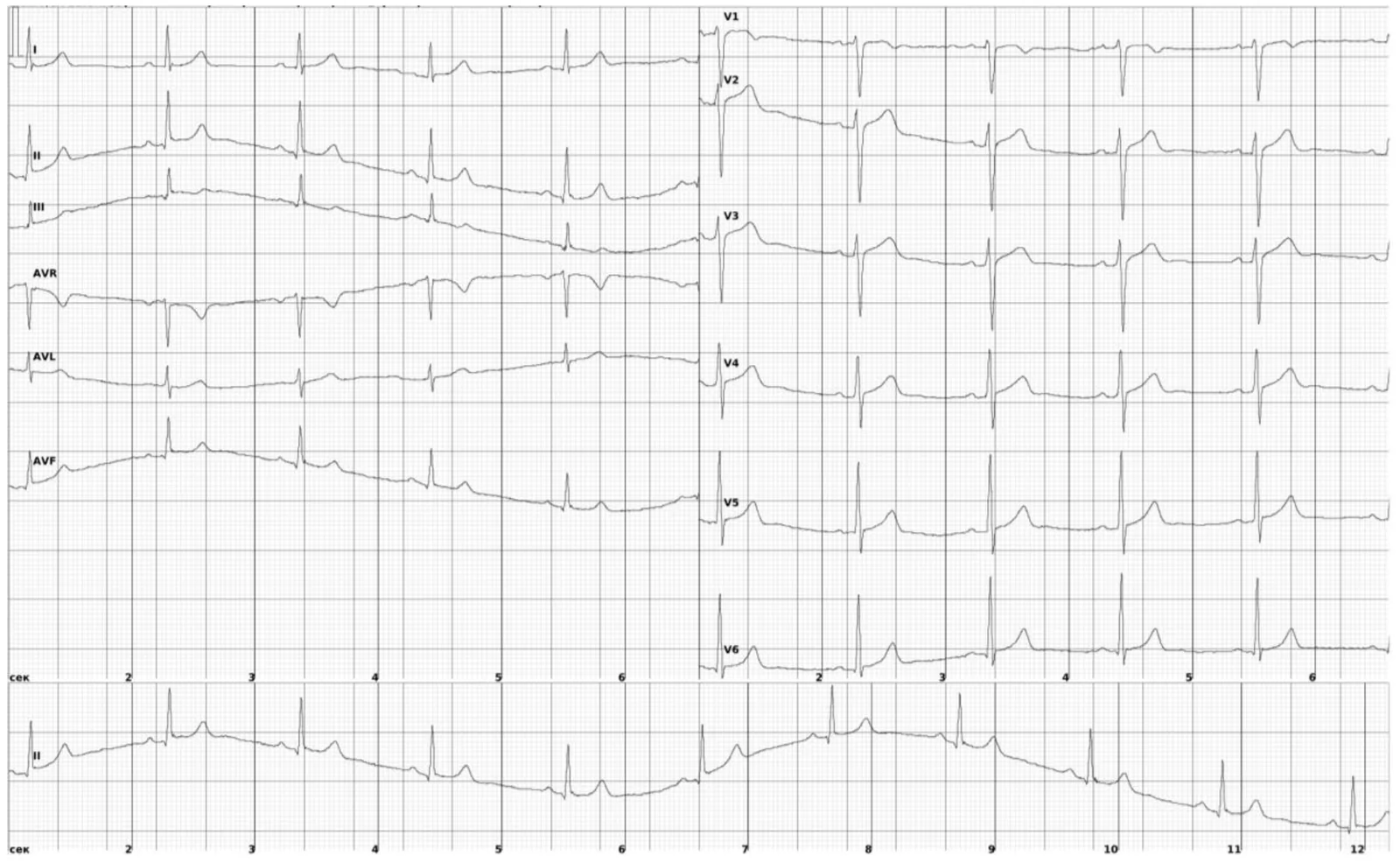
Resting electrocardiogram recorded eight years after cessation of anabolic-androgenic steroid use. Paper speed 25 mm/s, calibration 10 mm/mV (described in the text).

**Table 1:** Drug regimens of all four cycles, with cumulative doses.

Cycle	Agent	Route	Dose	Duration	Cumulative
2013 (5 months)	Testosterone esters	IM	350–500 mg/week	5 months	9.8 g
2014 (7 months)	Testosterone esters	IM	350–500 mg/week	7 months	13.2 g
	Boldenone undecylenate	IM	400–600 mg/week	4 months	8.7 g
	Drostanolone propionate	IM	300–700 mg/week	2 months	4.3 g
	Stanozolol	Oral	40 mg/day	8 weeks	2.2 g
	Stanozolol	IM	50 mg/day	4 weeks	1.4 g
	Oxandrolone	Oral	30 mg/day	10 weeks	2.1 g
	Somatropin	SC	10 IU/day	3 months	-
2015 (7 months)	Testosterone esters	IM	350–750 mg/week	7 months	15.4 g
	Boldenone undecylenate	IM	600–800 mg/week	4 months	12.2 g
	Drostanolone propionate	IM	300–700 mg/week	3 months	7.4 g
	Metenolone enanthate	IM	350 mg/week	2 months	3.0 g
	Stanozolol	Oral	40 mg/day	8 weeks	2.2 g
	Stanozolol	IM	50 mg/day	6 weeks	2.1 g
	Oxandrolone	Oral	30–50 mg/day	10 weeks	3.0 g
	Fluoxymesterone	Oral	5–10 mg/day	2 weeks	0.1 g
	Somatropin	SC	10 IU/day	3 months	-
2016–2017 (14 months)	Testosterone esters	IM	500–750 mg/week	14 months	34.8 g
	Boldenone undecylenate	IM	600–800 mg/week	6 months	19.1 g
	Drostanolone propionate	IM	300–700 mg/week	5 months	10.0 g
	Metenolone enanthate	IM	350 mg/week	3 months	4.6 g
	Stanozolol	Oral	40 mg/day	10 weeks	2.8 g
	Stanozolol	IM	50 mg/day	8 weeks	2.8 g
	Oxandrolone	Oral	30–50 mg/day	12 weeks	3.6 g
	Fluoxymesterone	Oral	5–20 mg/day	4 weeks	0.4 g
	Somatropin	SC	5–20 IU/day	6 months	-

Regimens were reconstructed from contemporaneous personal records retained by the patient. Doses and durations are known for each agent within each cycle, but the sequence in which agents were combined within a cycle could not be established. Oral and injectable stanozolol were used sequentially, never concurrently. Cumulative exposure to each agent over the four cycles was as follows: testosterone esters 73.0 g, boldenone undecylenate 40.0 g, drostanolone propionate 21.7 g, stanozolol 13.6 g, oxandrolone 8.7 g, metenolone enanthate 7.6 g and fluoxymesterone 0.5 g. Cumulative somatropin exposure was 12 months. IM, intramuscular; SC, subcutaneous.

**Table 2:** Serial laboratory findings during anabolic-androgenic steroid exposure and after cessation.

Parameter (Reference Range)	Dec 2015	Dec 2016	May 2017	Aug 2017	Oct 2017	Jul 2026
Total testosterone, nmol/L (8.9–42.0)	1.38	96.40	>120.00	-	>120.00	Normal
Free testosterone, pg/mL (1.00–28.28)	-	>100	-	-	>100	29.30
Dihydrotestosterone, pg/mL (143–842)	-	-	-	-	>2500	461.0
LH, mIU/mL (1.7–8.6)	<0.09	<0.09	<0.09	-	<0.09	7.2
FSH, mIU/mL (1.5–12.4)	-	0.05	<0.05	-	<0.05	5.75
SHBG, nmol/L (13–71)	-	4.1	5.5	2.5	2.2	37
IGF-1, ng/mL (121–336)	-	-	-	569.4	376.2	-
Estradiol, pmol/L (40–161)	44	<37	62	384	131	Normal
ALT, U/L (<41)	154	109	118	170	177	18.5
AST, U/L (<37–40)	92	65	73	126	85	34.5
HDL cholesterol, mmol/L	0.44	0.39	0.82	0.50	0.39	1.48
LDL cholesterol, mmol/L	4.84	3.11	5.19	3.11	3.20	1.80
Atherogenic index (2.0–3.0)	11.6	8.8	6.9	7.0	9.5	1.3
Hematocrit, % (39–49)	-	49.0	51.4	-	47.4	48.1
Hemoglobin, g/dL (13.2–17.3)	-	16.6	18.6	-	17.0	16.0
HOMA-IR (<2.7)	-	-	-	7.3	-	Insulin 7.7

Values in parentheses are laboratory reference ranges, which differ slightly between laboratories and assay platforms. December 2015 was recorded shortly after the end of a cycle, during the transient suppression of the hypothalamic-pituitary-gonadal axis that followed withdrawal; no post-cycle pharmacological therapy was used, and recovery occurred spontaneously. The four measurements between December 2016 and October 2017 were obtained during the final continuous cycle. The final column reports testing performed in July 2026, after the follow-up echocardiography. ALT, alanine aminotransferase; AST, aspartate aminotransferase; FSH, follicle-stimulating hormone; HDL, high-density lipoprotein; HOMA-IR, homeostatic model assessment of insulin resistance; IGF-1, insulin-like growth factor 1; LDL, low-density lipoprotein; LH, luteinizing hormone; SHBG, sex hormone-binding globulin.

**Table 3:** Concomitant agents other than anabolic-androgenic steroids used by the patient before presentation.

Agent (INN)	Route	Daily Dose
Somatropin (recombinant human growth hormone)	Subcutaneous	5–20 IU
Clenbuterol	Oral	20–40 µg
Yohimbine hydrochloride	Oral	5–20 mg
Creatine monohydrate	Oral	5 g

INN, international non-proprietary name; IU, international units.

**Table 4:** Comparison of baseline (2017) and follow-up (2025) transthoracic echocardiography.

Parameter	2017	2025	Change
**Chamber and wall dimensions**			
Aorta (annulus)	23 mm	22 mm	No meaningful change
Aorta (ascending)	26 mm	28 mm	No meaningful change
Left atrium (A4C)	38 × 46 mm	40 × 45 mm	No meaningful change
Right atrium (A4C)	50 × 57 mm	44 × 47 mm	Reduction in linear dimensions
LV end-diastolic diameter	49 mm	50 mm	No meaningful change
LV end-diastolic volume	n/a	91 mL	n/a
Interventricular septum (diastole)	22 mm	11.7 mm	−10.3 mm (47%)
LV posterior wall (diastole)	21 mm	10.8 mm	−10.2 mm (49%)
LV mass	551 g	214 g	−337 g (61%)
LV mass index *	261 g/m^2^	111 g/m^2^	−150 g/m^2^ (57%)
Relative wall thickness	0.86	0.43	−0.43 (marked regression; residual value above threshold)
Right ventricle (PLAX)	32 mm	29 mm	−3 mm
Right ventricular free wall	8.2 mm	3.9 mm	−4.3 mm (52%)
Main pulmonary artery	25 mm	22 mm	−3 mm
**LV systolic function**			
LVEF	90% †	75% ‡	Not comparable (different methods)
**LV diastolic function**			
Transmitral filling pattern	E/A below age-specific range	E/A within age-specific range	Change in filling pattern
E/A	1.18	1.45	Change in filling pattern

* LV mass index was calculated as LV mass divided by body surface area (DuBois formula): 2.11 m^2^ in 2017 and 1.92 m^2^ in 2025. Relative wall thickness above 0.42 indicates concentric geometry. † Teichholz method. ‡ Biplane Simpson method. A4C, apical four-chamber view; E/A, ratio of peak early (E) to late (A) transmitral filling velocity; LV, left ventricle; LVEF, left ventricular ejection fraction; n/a, not available; PLAX, parasternal long-axis view.

**Table 5:** Timeline of exposure, investigations and clinical course.

Date	Event	Investigations
2006	Began bodybuilding training, 3–6 resistance sessions per week	-
2013	First AAS cycle, 5 months (testosterone esters only)	-
2014	AAS cycle, 7 months; somatropin 10 IU/day for 3 months	-
2015	AAS cycle, 7 months; somatropin 10 IU/day for 3 months	-
Dec 2015	Shortly after end of cycle; transient suppression of the gonadal axis, no post-cycle therapy	Testosterone 1.38 nmol/L, LH <0.09 mIU/mL, ALT 154 U/L
2016–2017	Continuous 14-month cycle (Table 1), with somatropin for 6 months in 2017	-
Dec 2016	During cycle	Testosterone 96.40 nmol/L, gonadotrophins suppressed, HDL 0.39 mmol/L
May 2017	During cycle	Testosterone >120 nmol/L, LDL 5.19 mmol/L, ALT 118 U/L
Aug 2017	During cycle	IGF-1 569.4 ng/mL, HOMA-IR 7.3, estradiol 384 pmol/L
Oct 2017	During cycle, 2 months before presentation	IGF-1 376.2 ng/mL, DHT >2500 pg/mL, ALT 177 U/L
Dec 2017	Onset of palpitations; first cardiology assessment. BP 140/85 mm Hg. ECG: sinus rhythm, ventricular ectopic beats, voltage criteria for LV hypertrophy	Baseline echocardiography (Table 4): IVS 22 mm, PW 21 mm, RV free wall 8.2 mm
Dec 2017	Complete cessation of AAS, somatropin, clenbuterol and yohimbine. Observation elected; no cardiovascular pharmacotherapy started. Competitive bodybuilding discontinued	-
Dec 2017–Mar 2018	Spontaneous resolution of palpitations without antiarrhythmic treatment	-
2018–2025	Resistance training continued at reduced volume and intensity; aerobic exercise added; gradual 20 kg reduction in body weight at unchanged body fat percentage. No echocardiography performed during this interval	Annual resting 12-lead ECG only
Until 2020	ECG voltage criteria for hypertrophy present	Annual resting 12-lead ECG only
After 2020	ECG voltage criteria no longer present	Annual resting 12-lead ECG only
Oct 2025	Reassessment at 8 years. Asymptomatic. BP 120/75 mm Hg. ECG normal	Follow-up echocardiography (Table 4): IVS 11.7 mm, PW 10.8 mm, RV free wall 3.9 mm; Figure 1
Jul 2026	Laboratory reassessment	Gonadal axis, lipids, aminotransferases and insulin sensitivity normalized (Table 2)

The eight-year interval between the two echocardiographic examinations, during which only annual resting electrocardiography was performed, is shown explicitly. ALT, alanine aminotransferase; BP, blood pressure; DHT, dihydrotestosterone; ECG, electrocardiogram; HDL, high-density lipoprotein; HOMA-IR, homeostatic model assessment of insulin resistance; IGF-1, insulin-like growth factor 1; IVS, interventricular septum; LDL, low-density lipoprotein; LH, luteinizing hormone; PW, posterior wall; RV, right ventricle.

**Table 6:** Key studies of cardiac structure and function in anabolic-androgenic steroid users.

Study (Year)	Design, *n*	AAS Exposure	Main Findings	Evidence on Reversibility
Smit et al., HAARLEM (2021) [3]	Prospective cohort, *n* = 100 (echocardiography in 31)	16-week cycle	LV mass +28.3 g; LVEF −4.9%; E/A −0.45	Complete normalization of all parameters ~8 months after cessation
Baggish et al. (2017) [4]	Cross-sectional, *n* = 140	≥2 years	LVEF 52% vs. 63%; increased coronary plaque volume	Some abnormalities persisted after cessation
Abdullah et al. (2024) [11]	Cross-sectional, *n* = 101	11 ± 7 years	LVEF 49 ± 7% vs. 59 ± 5%; biventricular dysfunction	Dysfunction persisted in former users at 6 ± 6 years
Bulut et al. (2024) [12]	Cross-sectional, ^82^Rb positron emission tomography	Long-term	Reduced myocardial flow reserve	Microvascular dysfunction persists for years
Windfeld-Mathiasen et al. (2025) [7]	Registry, *n* = 1189 vs. 59,450	Not specified	Cardiomyopathy aHR 8.90 (4.99–15.88) over ~11 years	Not assessed
Vecchiato et al. (2025) [6]	Cohort, *n* = 20,286	Not specified	Mortality 81/100,000 person-years; sudden cardiac death 38% of deaths	Not assessed
Fyksen et al. (2025) [9]	Prospective cohort, 16-year follow-up	>10 years in some	Continuing users retained increased LV mass and reduced LVEF	In those who stopped, LV mass similar to strength-trained non-users; LVEF appeared to improve
Buhl et al., FIDO-DK (2025) [10]	Cross-sectional	5-year threshold	Reduced LV and right ventricular GLS; higher coronary calcium score (OR 1.23 per year of use)	Myocardial dysfunction less pronounced in former than current users
Cavalcante et al. (2026) [8]	Systematic review and meta-analysis, 35 studies, *n* ≈ 2000	Variable	LVEF −2.25%; GLS −3.34%; septal thickness +1.24 mm	Not assessed

AAS, anabolic-androgenic steroids; GLS, global longitudinal strain; LV, left ventricular; LVEF, left ventricular ejection fraction; OR, odds ratio.

**Table 7:** Published cases of anabolic-androgenic steroid-associated cardiomyopathy compared with the present case.

Author (Year)	Age, Sex	AAS Exposure	Baseline Findings	Heart Failure Therapy	Follow-Up	Outcome
Joseph et al. (2017) [15]	Male	Not specified	Reduced LVEF	Yes	Months	Recovery of myocardial function
Angelini et al. (2019) [16]	31, male	~12 years	Dilated cardiomyopathy, LVEF 23%	Yes (OMT)	Not specified	Irreversible dilated cardiomyopathy
Gul and Shahid (2022) [14]	Young female	Not specified	Heart failure symptoms	Yes	Months	Resolution of symptoms
Milevski et al. (2022) [13]	46, male	Long-term	Dilated cardiomyopathy, LVEF 12%	Yes (OMT)	Months	Near-complete recovery of LVEF
Charaya et al. (2024) [18]	49, male	>4 years	Heart failure, LVEF 20%, LV hypertrophy	Yes (OMT)	Several years	LVEF recovered to 62%, then relapsed to 31% after AAS were resumed
Present case	26 – 34, male	33 months	Concentric LV hypertrophy, increased RV free wall thickness; E/A 1.18	No	8 years	Marked regression of hypertrophy; E/A rose to 1.45

AAS, anabolic-androgenic steroids; LV, left ventricular; LVEF, left ventricular ejection fraction; OMT, optimal medical therapy.

## Data Availability

The de-identified data underlying this report are available from the corresponding author on reasonable request.

## Abbreviations

4E-BP1	eukaryotic initiation factor 4E-binding protein 1
8-OHdG	8-hydroxy-2′-deoxyguanosine
A4C	apical four-chamber view
AAS	anabolic-androgenic steroids
ALT	alanine aminotransferase
ANP	atrial natriuretic peptide
AR	androgen receptor
AST	aspartate aminotransferase
AT1R	angiotensin II type 1 receptor
BP	blood pressure
CARE	CAse REport guidelines
CI	confidence interval
DHT	dihydrotestosterone
E/A	ratio of peak early (E) to late (A) transmitral filling velocity
eNOS	endothelial nitric oxide synthase
ERK1/2	extracellular signal-regulated kinase 1/2
FSH	follicle-stimulating hormone
GLS	global longitudinal strain
HDL	high-density lipoprotein
HOMA-IR	homeostatic model assessment of insulin resistance
ICAM-1	intercellular adhesion molecule 1
IGF-1	insulin-like growth factor 1
IKr, IKs	rapid and slow components of the delayed rectifier potassium current
IM	intramuscular
INN	international non-proprietary name
IP3	inositol 1,4,5-trisphosphate
IU	international units
LDL	low-density lipoprotein
IVS	interventricular septum
LH	luteinizing hormone
LV	left ventricular/left ventricle
LVEDD	left ventricular end-diastolic diameter
LVEF	left ventricular ejection fraction
MAPK	mitogen-activated protein kinase
mTOR	mammalian target of rapamycin
NF-κB	nuclear factor kappa B
NLRP3	NOD-like receptor family pyrin domain containing 3
NO	nitric oxide
OMT	optimal medical therapy
OR	odds ratio
PI3K	phosphatidylinositol 3-kinase
PLAX	parasternal long-axis view
PW	left ventricular posterior wall
rhGH	recombinant human growth hormone
ROS	reactive oxygen species
S6K1	40S ribosomal protein S6 kinase 1
SC	subcutaneous
SHBG	sex hormone-binding globulin
TAPSE	tricuspid annular plane systolic excursion
TLR4	Toll-like receptor 4
TUNEL	terminal deoxynucleotidyl transferase dUTP nick end labeling
VCAM-1	vascular cell adhesion molecule 1
